# Supplementary material for: Decreases and Pronounced Geographic Variability in Antibiotic Prescribing in Medicaid
Source: Pharmacy (Basel). 2024 Mar 1;12(2):46. doi: 10.3390/pharmacy12020046 (PMC10961814; doi:10.3390/pharmacy12020046)
Supplement: Supplementary file 1 [file pharmacy-12-00046-s001.zip › pharmacy-2764934-supplementary.pdf]

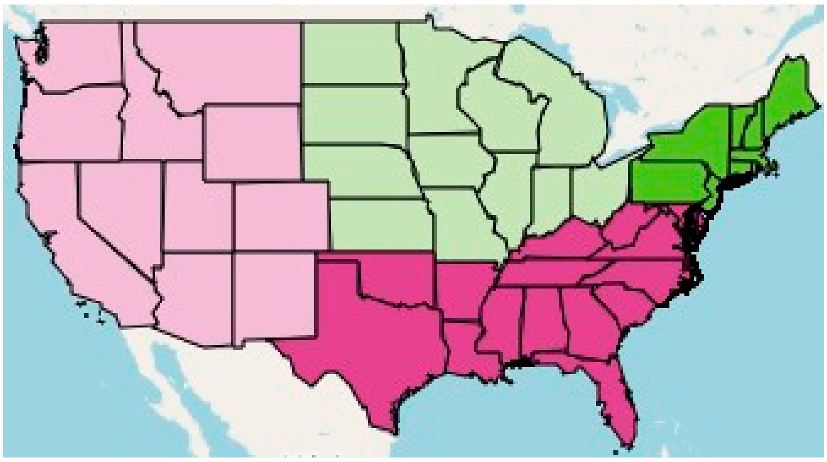

Supplemental Figure S1. US Census regions including the West, Midwest, South, and Northeast. Alaska and Hawaii are not shown. Image created with http://www.heatmapper.ca/geomap/.
